# 2-Amino-2,3-dimethyl­butanamide

**DOI:** 10.1107/S1600536810015679

**Published:** 2010-05-08

**Authors:** Yongbiao Yin

**Affiliations:** a Department of Chemistry, Mudanjiang Teachers College, Mudanjiang 157012, People’s Republic of China

## Abstract

The title compound, C_6_H_14_N_2_O, was synthesized by the reaction between 2-amino-2,3-dimethyl­butanonitrile and oil of vitriol (sulfuric acid). A racemic mixture of *L*- and *R*-2-amino-2,3-di­methyl­butanamide was characterized crystallographically. In the crystal structure, inter­molecular N—H⋯O hydrogen bonds link the two enanti­omers into a three-dimensional network.

## Related literature

2-Amino-2,3-dimethyl­butanamide, a common inter­mediate in the synthesis of imidazolinone compounds, is an excellent weedicide, usually used as racemic mixture of the levo and dextral enanti­omers, see: Goatz *et al.* (1990 ▶); Harir *et al.* (2007 ▶).
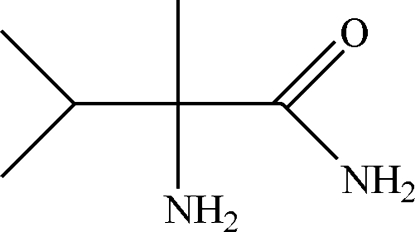


## Experimental

### 

#### Crystal data

C_6_H_14_N_2_O
*M* *_r_* = 130.19
Monoclinic, 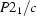

*a* = 12.1766 (8) Å
*b* = 6.1741 (4) Å
*c* = 10.2322 (5) Å
β = 94.682 (6)°
*V* = 766.69 (8) Å^3^
*Z* = 4
Mo *K*α radiation
μ = 0.08 mm^−1^
*T* = 120 K
0.14 × 0.11 × 0.10 mm

#### Data collection

Bruker SMART APEXII CCD area-detector diffractometer
Absorption correction: multi-scan (*SADABS*; Bruker, 2005 ▶) *T*_min_ = 0.991, *T*_max_ = 0.993
3390 measured reflections
1503 independent reflections
922 reflections with *I* > 2σ(*I*)
*R*_int_ = 0.024

#### Refinement

*R*[*F* ^2^ > 2σ(*F* ^2^)] = 0.049
*wR*(*F* ^2^) = 0.152
*S* = 0.95
1503 reflections
86 parameters
H-atom parameters constrained
Δρ_max_ = 0.24 e Å^−3^
Δρ_min_ = −0.17 e Å^−3^

### 

Data collection: *APEX2* (Bruker, 2005 ▶); cell refinement: *SAINT* (Bruker, 2005 ▶); data reduction: *SAINT*; program(s) used to solve structure: *SHELXS97* (Sheldrick, 2008 ▶); program(s) used to refine structure: *SHELXL97* (Sheldrick, 2008 ▶); molecular graphics: *ORTEP-3 for Windows* (Farrugia, 1997 ▶); software used to prepare material for publication: *WinGX* (Farrugia, 1999 ▶).

## Supplementary Material

Crystal structure: contains datablocks I, global. DOI: 10.1107/S1600536810015679/jh2145sup1.cif

Structure factors: contains datablocks I. DOI: 10.1107/S1600536810015679/jh2145Isup2.hkl

Additional supplementary materials:  crystallographic information; 3D view; checkCIF report

## Figures and Tables

**Table 1:** Hydrogen-bond geometry (Å, °)

*D*—H⋯*A*	*D*—H	H⋯*A*	*D*⋯*A*	*D*—H⋯*A*
N2—H2*B*⋯O1^i^	0.89	2.52	3.364 (2)	158
N1—H1*B*⋯O1^i^	0.86	2.19	3.0295 (19)	165
N1—H1*A*⋯O1^ii^	0.86	2.20	3.054 (2)	176
N2—H2*C*⋯O1^iii^	0.89	2.51	3.393 (3)	172

Symmetry codes: (i) 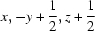; (ii) 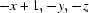; (iii) 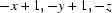.

**Fig. 1.**
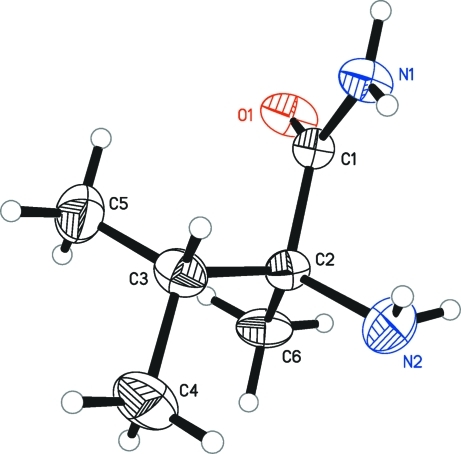
The molecular structure with atom labels and 30% probability displacement ellipsoids for non-H atoms.

**Fig. 2.**
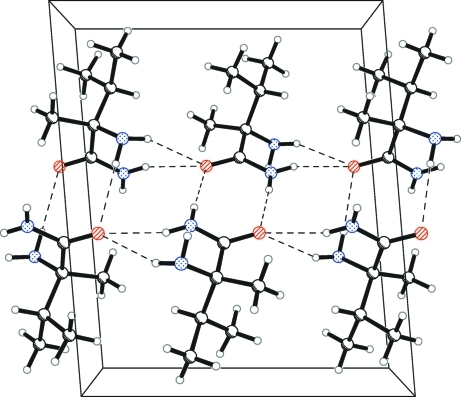
The packing diagram of molecules, viewed down the b axis, with the weak interactions shown as dashed lines.

## supplementary crystallographic information

### Comment

2-Amino-2,3-dimethylbutanamide is common intermediate in the synthesis of imidazolinone compounds, excellent weedicide, usually used as racemic mixture of the levo and dextral enantiomers (Goatz, *et al.*, 1990; Harir, *et al.*, 2007). We report herein the synthesis and the structural determination of the title molecule. The as synthesis compound contains the both chiral components of *L*- and *R*-2-amino-2,3-dimethylbutanamide, and as a consequence, the space group of crystal is centrosymmetric P2_1_/c which contain gliding plane and center of symmetry. In addition, intermolecular N–H···O hydrogen bonds linked the two enantiomers into unlimited three dimensional network.

### Experimental

2-Amino-2,3-dimethylbutanenitrile liquid (46.7 g, 0.417 mol) was added to the oil of vitriol solution (104.2 ml) under N_2_ protection in cold water bath. Next, the solution along with the white solid appeared was slowly poured into 150 grams of ice water after three days of stir at room temperature. Then, Na_2_CO_3_ (221 g) and 50% NaOH (38 ml) were consumed to basify the solution to pH value 9.0, giving rise to plenty of white solid. The title compound (25.8 g) with a m.p of 76-80 degrees, yielding 47.6% was obtained after filtration and purification through extraction with dichloromethane. The suitable single crystals for X-ray diffraction was from slow evaporation of solvent from the title compound dichloromethane solution.

### Refinement

H atoms bonded to O atom of free water molecule were located in a difference map. All the other H atoms were placed in calculated positions and refined as riding, with C–H = 0.96–0.98 Å, and O–H = 0.85 Å, and *U*_iso_(H) = 1.2 or 1.5*U*_eq_(C,*O*).

### Crystal data

**Table:**

C_6_H_14_N_2_O	*F*(000) = 288.0
*M**_r_* = 130.19	*D*_x_ = 1.128 Mg m^−^^3^
Monoclinic, *P*2_1_/*c*	Mo *K*α radiation, λ = 0.71073 Å
Hall symbol: -P 2ybc	Cell parameters from 3786 reflections
*a* = 12.1766 (8) Å	θ = 3.4–29.4°
*b* = 6.1741 (4) Å	µ = 0.08 mm^−^^1^
*c* = 10.2322 (5) Å	*T* = 120 K
β = 94.682 (6)°	Prism, colourless
*V* = 766.69 (8) Å^3^	0.14 × 0.11 × 0.10 mm
*Z* = 4	

### Data collection

**Table:**

Bruker SMART APEXII CCD area-detector diffractometer	1503 independent reflections
Radiation source: fine-focus sealed tube	922 reflections with *I* > 2σ(*I*)
graphite	*R*_int_ = 0.024
φ and ω scans	θ_max_ = 26.0°, θ_min_ = 3.4°
Absorption correction: multi-scan (*SADABS*; Bruker, 2005)	*h* = −15→15
*T*_min_ = 0.991, *T*_max_ = 0.993	*k* = −7→6
3390 measured reflections	*l* = −8→12

### Refinement

**Table:**

Refinement on *F*^2^	Primary atom site location: structure-invariant direct methods
Least-squares matrix: full	Secondary atom site location: difference Fourier map
*R*[*F*^2^ > 2σ(*F*^2^)] = 0.049	Hydrogen site location: inferred from neighbouring sites
*wR*(*F*^2^) = 0.152	H-atom parameters constrained
*S* = 0.95	*w* = 1/[σ^2^(*F*_o_^2^) + (0.0943*P*)^2^] where *P* = (*F*_o_^2^ + 2*F*_c_^2^)/3
1503 reflections	(Δ/σ)_max_ = 0.001
86 parameters	Δρ_max_ = 0.24 e Å^−^^3^
0 restraints	Δρ_min_ = −0.16 e Å^−^^3^

### Special details

**Table:**

Geometry. All esds (except the esd in the dihedral angle between two l.s. planes) are estimated using the full covariance matrix. The cell esds are taken into account individually in the estimation of esds in distances, angles and torsion angles; correlations between esds in cell parameters are only used when they are defined by crystal symmetry. An approximate (isotropic) treatment of cell esds is used for estimating esds involving l.s. planes.
Refinement. Refinement of *F*^2^ against ALL reflections. The weighted *R*-factor wR and goodness of fit *S* are based on *F*^2^, conventional *R*-factors *R* are based on *F*, with *F* set to zero for negative *F*^2^. The threshold expression of *F*^2^ > σ(*F*^2^) is used only for calculating *R*-factors(gt) etc. and is not relevant to the choice of reflections for refinement. *R*-factors based on *F*^2^ are statistically about twice as large as those based on *F*, and *R*- factors based on ALL data will be even larger.

### Fractional atomic coordinates and isotropic or equivalent isotropic displacement parameters (Å^2^)

**Table:**

	*x*	*y*	*z*	*U*_iso_*/*U*_eq_	
N1	0.57193 (13)	0.1711 (3)	0.13703 (14)	0.0498 (5)	
H1A	0.5245	0.0685	0.1239	0.060*	
H1B	0.5902	0.2155	0.2155	0.060*	
O1	0.59128 (12)	0.1981 (2)	−0.07665 (11)	0.0601 (5)	
C1	0.61676 (14)	0.2604 (3)	0.03676 (16)	0.0387 (5)	
C2	0.70199 (14)	0.4409 (3)	0.06667 (15)	0.0411 (5)	
C3	0.81019 (15)	0.3346 (3)	0.12505 (19)	0.0550 (6)	
H3	0.7932	0.2593	0.2053	0.066*	
C4	0.89925 (19)	0.5008 (4)	0.1643 (3)	0.0846 (8)	
H4A	0.9636	0.4281	0.2029	0.127*	
H4B	0.8726	0.6000	0.2267	0.127*	
H4C	0.9176	0.5791	0.0880	0.127*	
C5	0.8548 (2)	0.1667 (4)	0.0355 (3)	0.0873 (9)	
H5A	0.8749	0.2356	−0.0432	0.131*	
H5B	0.7993	0.0593	0.0136	0.131*	
H5C	0.9185	0.0988	0.0793	0.131*	
C6	0.71901 (18)	0.5628 (3)	−0.05696 (19)	0.0570 (6)	
H6A	0.6504	0.6252	−0.0912	0.086*	
H6B	0.7454	0.4653	−0.1205	0.086*	
H6C	0.7721	0.6757	−0.0382	0.086*	
N2	0.66166 (17)	0.5932 (3)	0.16527 (19)	0.0751 (6)	
H2B	0.6624	0.5270	0.2426	0.113*	
H2C	0.5931	0.6334	0.1394	0.113*	

### Atomic displacement parameters (Å^2^)

**Table:**

	*U*^11^	*U*^22^	*U*^33^	*U*^12^	*U*^13^	*U*^23^
N1	0.0592 (10)	0.0583 (9)	0.0318 (8)	−0.0172 (8)	0.0032 (7)	0.0000 (7)
O1	0.0751 (10)	0.0746 (9)	0.0299 (7)	−0.0280 (7)	0.0000 (6)	−0.0034 (6)
C1	0.0427 (9)	0.0431 (9)	0.0297 (9)	−0.0011 (7)	−0.0004 (7)	0.0004 (7)
C2	0.0506 (10)	0.0412 (9)	0.0314 (9)	−0.0036 (8)	0.0022 (8)	−0.0013 (7)
C3	0.0506 (12)	0.0607 (12)	0.0521 (12)	−0.0076 (9)	−0.0044 (10)	0.0087 (10)
C4	0.0588 (13)	0.0998 (18)	0.0922 (18)	−0.0196 (13)	−0.0122 (13)	−0.0087 (15)
C5	0.0627 (14)	0.0735 (15)	0.124 (2)	0.0132 (12)	−0.0037 (15)	−0.0172 (15)
C6	0.0761 (13)	0.0509 (11)	0.0432 (11)	−0.0189 (10)	−0.0011 (10)	0.0130 (9)
N2	0.0845 (14)	0.0712 (12)	0.0707 (12)	0.0004 (10)	0.0127 (11)	−0.0231 (10)

### Geometric parameters (Å, °)

**Table:**

N1—C1	1.321 (2)	C4—H4A	0.9600
N1—H1A	0.8600	C4—H4B	0.9600
N1—H1B	0.8600	C4—H4C	0.9600
O1—C1	1.2377 (18)	C5—H5A	0.9600
C1—C2	1.536 (2)	C5—H5B	0.9600
C2—N2	1.492 (2)	C5—H5C	0.9600
C2—C6	1.501 (2)	C6—H6A	0.9600
C2—C3	1.548 (2)	C6—H6B	0.9600
C3—C5	1.513 (3)	C6—H6C	0.9600
C3—C4	1.523 (3)	N2—H2B	0.8900
C3—H3	0.9800	N2—H2C	0.8900
			
C1—N1—H1A	120.0	H4A—C4—H4B	109.5
C1—N1—H1B	120.0	C3—C4—H4C	109.5
H1A—N1—H1B	120.0	H4A—C4—H4C	109.5
O1—C1—N1	120.70 (16)	H4B—C4—H4C	109.5
O1—C1—C2	121.70 (15)	C3—C5—H5A	109.5
N1—C1—C2	117.59 (13)	C3—C5—H5B	109.5
N2—C2—C6	109.24 (16)	H5A—C5—H5B	109.5
N2—C2—C1	109.75 (15)	C3—C5—H5C	109.5
C6—C2—C1	109.47 (13)	H5A—C5—H5C	109.5
N2—C2—C3	108.86 (13)	H5B—C5—H5C	109.5
C6—C2—C3	111.48 (16)	C2—C6—H6A	109.5
C1—C2—C3	108.02 (14)	C2—C6—H6B	109.5
C5—C3—C4	109.74 (19)	H6A—C6—H6B	109.5
C5—C3—C2	113.15 (15)	C2—C6—H6C	109.5
C4—C3—C2	112.42 (17)	H6A—C6—H6C	109.5
C5—C3—H3	107.1	H6B—C6—H6C	109.5
C4—C3—H3	107.1	C2—N2—H2B	109.4
C2—C3—H3	107.1	C2—N2—H2C	109.1
C3—C4—H4A	109.5	H2B—N2—H2C	109.5
C3—C4—H4B	109.5		
			
O1—C1—C2—N2	136.55 (18)	N2—C2—C3—C5	176.71 (19)
N1—C1—C2—N2	−44.2 (2)	C6—C2—C3—C5	−62.7 (2)
O1—C1—C2—C6	16.7 (2)	C1—C2—C3—C5	57.6 (2)
N1—C1—C2—C6	−164.08 (17)	N2—C2—C3—C4	−58.3 (2)
O1—C1—C2—C3	−104.90 (19)	C6—C2—C3—C4	62.3 (2)
N1—C1—C2—C3	74.3 (2)	C1—C2—C3—C4	−177.40 (17)

### Hydrogen-bond geometry (Å, °)

**Table:**

*D*—H···*A*	*D*—H	H···*A*	*D*···*A*	*D*—H···*A*
N2—H2B···O1^i^	0.89	2.52	3.364 (2)	158
N1—H1B···O1^i^	0.86	2.19	3.0295 (19)	165
N1—H1A···O1^ii^	0.86	2.20	3.054 (2)	176
N2—H2C···O1^iii^	0.89	2.51	3.393 (3)	172

Symmetry codes: (i) *x*, −*y*+1/2, *z*+1/2; (ii) −*x*+1, −*y*, −*z*; (iii) −*x*+1, −*y*+1, −*z*.
